# A Segmentation of Melanocytic Skin Lesions in Dermoscopic and Standard Images Using a Hybrid Two-Stage Approach

**DOI:** 10.1155/2021/5562801

**Published:** 2021-04-06

**Authors:** Yoo Na Hwang, Min Ji Seo, Sung Min Kim

**Affiliations:** ^1^Department of Medical Biotechnology, Dongguk University-Bio Medi Campus, 10326, Republic of Korea; ^2^Department of Medical Device Industry, Dongguk University, 04620, Republic of Korea

## Abstract

The segmentation of a skin lesion is regarded as very challenging because of the low contrast between the lesion and the surrounding skin, the existence of various artifacts, and different imaging acquisition conditions. The purpose of this study is to segment melanocytic skin lesions in dermoscopic and standard images by using a hybrid model combining a new hierarchical *K*-means and level set approach, called HK-LS. Although the level set method is usually sensitive to initial estimation, it is widely used in biomedical image segmentation because it can segment more complex images and does not require a large number of manually labelled images. The preprocessing step is used for the proposed model to be less sensitive to intensity inhomogeneity. The proposed method was evaluated on medical skin images from two publicly available datasets including the PH^2^ database and the Dermofit database. All skin lesions were segmented with high accuracies (>94%) and Dice coefficients (>0.91) of the ground truth on two databases. The quantitative experimental results reveal that the proposed method yielded significantly better results compared to other traditional level set models and has a certain advantage over the segmentation results of U-net in standard images. The proposed method had high clinical applicability for the segmentation of melanocytic skin lesions in dermoscopic and standard images.

## 1. Introduction

Melanoma is a dangerous skin cancer that mostly appears in pigmented cells (melanocytes) in the skin. It is a major cause of death associated with skin cancer [1]. Early diagnosis of melanoma is essential because early-stage detection and proper treatment increase the survival rate [2, 3]. Melanoma is mostly detected by expert dermatologists through visual inspection using the naked eye alone with a diagnostic accuracy of about 60% [4, 5].

Clinical images are normally obtained using digital cameras. However, the imaging conditions are frequently inconsistent because images are acquired from different distances or under variable illumination conditions. These may lead to problems when the size of the lesion is too small. Dermoscopy, a technique whereby a hand-held device is used to detect a mole and inspect the underlying skin, is better than unaided visual inspection and increases the sensitivity of detection by 10-30% [6]. Nevertheless, the within- and between-observer concordance is very low, even for expert clinicians [7]. An additional problem is related to the presence of intrinsic noise and artifacts, such as hair, blood vessels, air bubbles, and frames; variegated colors inside the lesion; and the lack of distinct boundaries to the surrounding skin [8]. These make it difficult to distinguish the skin lesion [9]. Thus, a growing interest has developed in the computational analysis of skin lesion images to assist clinicians in distinguishing early melanoma from benign lesions [10].

The first step in the computerized analysis of skin lesion images is the segmentation of the lesion. The segmentation of skin lesions from the surrounding skin is essential to provide important information for an accurate analysis of skin lesions and to extract important clinical features such as atypical pigment networks, blue-white areas, and globules [11, 12]. Moreover, this step is the key process by which lesion diameters are quantified and the extent of border irregularities are evaluated. Effective methods have been proposed to improve the segmentation accuracy.

Active contour-based medical image segmentation, such as a level set, is a well-established approach [13]. It was first introduced by Osher and Sethian. Level set evolution, which is established on partial differential equations and dynamic implicit interfaces, has been widely used in the field of medical image segmentation. Silveira and Marquez [13], Nourmohamadi and Pourghassem [14], and Li et al. [15] used the level set method with clustering-based initial estimation models, such as the Otsu thresholding, weighting combination of fuzzy C-mean and *K*-means, and spatial fuzzy clustering. The level set method is an efficient way to identify low contrast boundaries [16]. Schmid [17] presented a color clustering-based technique with a modified version of fuzzy C-means clustering. Donadey et al. [18] also detected a border by using the intensity component of hue-saturation-intensity (HSI) space. However, traditional models such as the region-based active contour model often failed when applied to images containing inhomogeneities. These are very sensitive to parameter tuning [16]. Recently, machine learning algorithms, including deep learning architectures, such as Residual net [1] or U-net [9], have emerged as reliable segmentation methods for skin lesion images. However, these algorithms can deal with inhomogeneities but require postprocessing and a large training set [16]. Some cases still show a low performance of skin lesion segmentation due to very low contrast and hair artifacts in skin lesion images [8]. These make it hard to train effectively deep networks with a large number of parameters [1].

To tackle the abovementioned problems, a hybrid model which integrates unsupervised learning with a region-based active contour model is proposed in this study. The proposed method combined the hierarchical *K*-means clustering and level set methods. This model thus can be less sensitive to parameter controlling of the level set model and to intensity inhomogeneity. The rest of this study was organized as follows.  Section 2 introduces the overall processes used in the segmentation: (a) preprocessing, (b) segmentation, and (c) performance evaluation. Sections 3 and 4 provide the experimental results and discussions, respectively. Finally, Section 5 concluded the paper and identified future directions.

## 2. Materials and Methods

**Figure 1:**
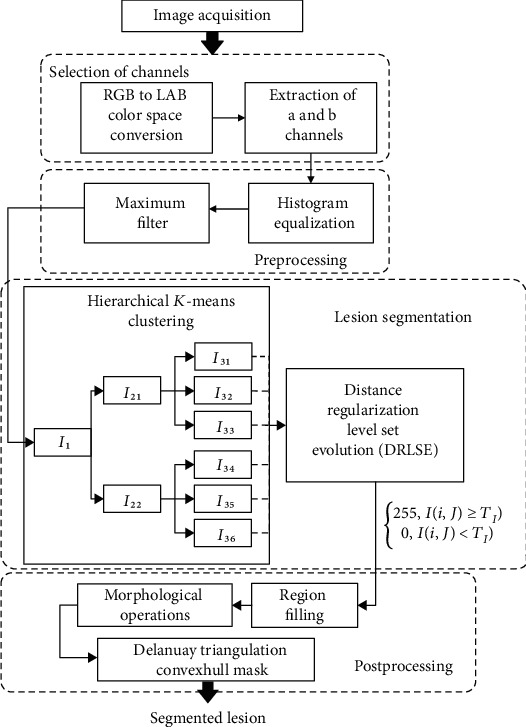
Overall flowchart of the proposed scheme for the segmentation of each skin lesion image in dermoscopic and standard images.

To segment a melanocytic skin lesion accurately, the proposed method was implemented through four steps: image acquisition, preprocessing, a two-stage segmentation model, and postprocessing. The statistical significance of the suggested method was evaluated by the Jaccard index, the Dice coefficient, sensitivity, and other measures.  Figure 1 shows an overall flowchart of the suggested approach for the segmentation of each skin lesion. The detailed procedures are described below.

### 2.1. Image Acquisition

This study used dermoscopic and standard images from the following two dermatology atlases:

1. The PH^2^ data [19] is a dataset that includes 200 dermoscopic images, including 40 malignant melanomas and 160 melanocytic nevus (80 common nevi and 80 atypical nevi) at 768 × 560 resolution, collected by a group of researchers from the Technical Universities of Porto and Lisbon in the Dermatology Service of Pedro Hispano Hospital. Each image has 8-bit red, green, and blue (RGB) channels.
2. The Edinburgh Dermofit Image Library [20] is a dataset that includes high-quality skin lesion images (1,300 biopsy-proven cancers and moles) collected across 10 different classes, including 331 melanocytic nevus images and 76 malignant melanoma images. The images are snapshots of the skin lesions surrounded by normal skin captured using a Canon EOS 350D SLR camera with a pixel resolution of about 0.03 mm.

**Figure 2:**
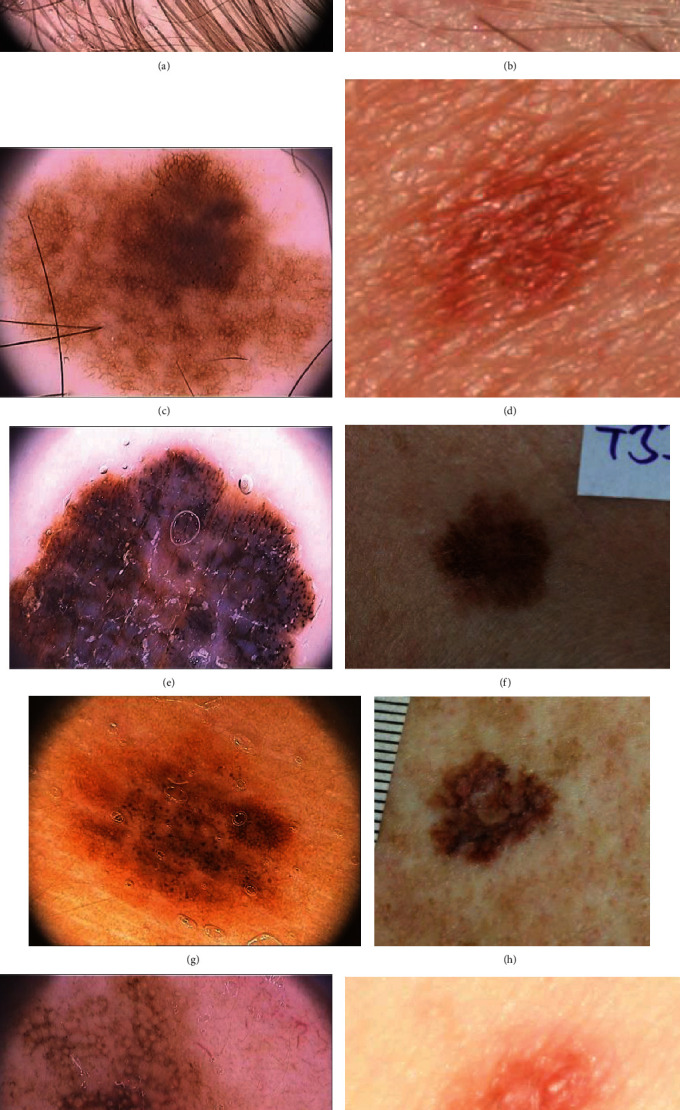
Illustrative examples of dermoscopic images (a, c, e, g, and i) and standard images (b, d, f, h, and j).

Figure 2 shows the sample images with different artifacts and aberrations. The skin images obtained from these atlases were annotated by expert dermatology resource providers. All images were allocated to diagnosis labels and binary segmentation masks that denote the lesion area. In the binary segmentation mask, the pixels outside the lesions were assigned pixel intensity values of 0 and pixels inside the lesion were assigned pixel intensity values of 255. 116 images of malignant melanoma and 491 images of melanocytic nevus were acquired from two different atlases (Table 1).

### 2.2. Preprocessing

**Figure 3:**
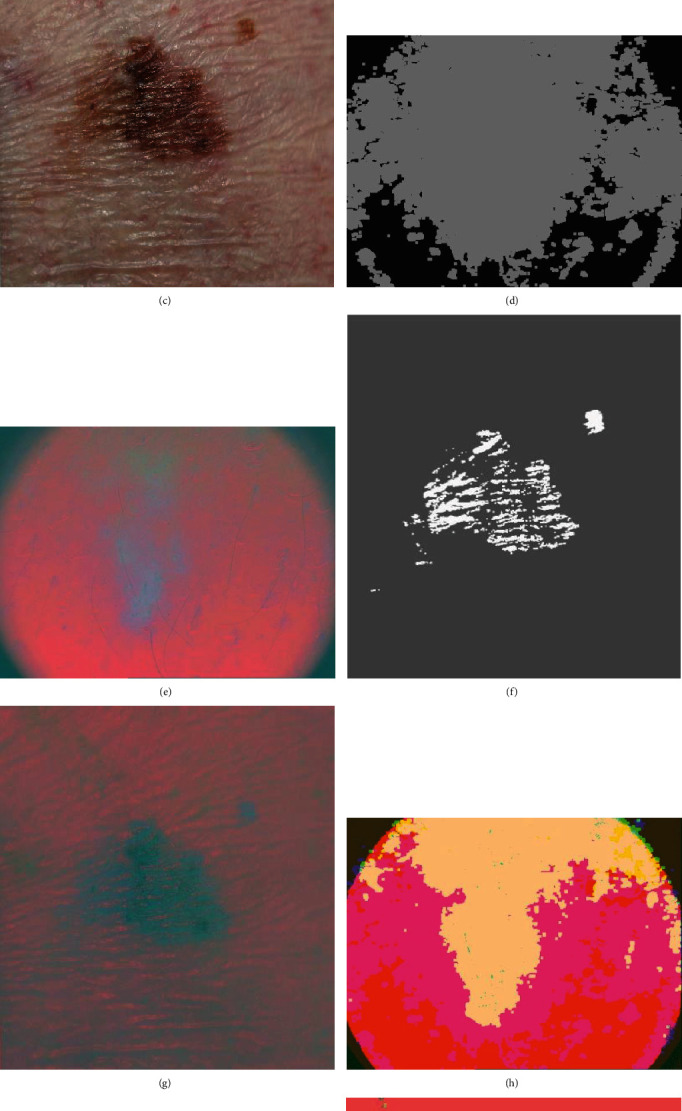
Example results of preprocessing on PH^2^ (a, c, e, g, and i) and Dermofit (b, d, f, h, and j). The first row (a, b) contains original images. The second row (c, d) contains the images converted from RGB to CIE LAB color space. The 3rd row (e, f) contains histogram equalization images of channel *a* and (g, h) channel *b*. The 4th row (i, j) contains the final fusion images.

Dermoscopic and standard images usually contain artifacts such as illumination variations, dermoscopic gel, air bubbles, and outlines (hair, skin lines, vignetting around the lesion, ruler markers, and blood vessels). These artifacts can attenuate the accuracy of border detection and increase computational time. As a result, there is a need for robust methods to attenuate artifacts. To do this, the first step of this study is to create an image that converts the image into a different color space and removes artifacts including hair, vignetting around the lesion, and ruler markings as shown in Figure 3.

All skin images are RGB-colored images, which are the combination of gray values from the individual R, G, and B channels [21]. This color space is not as sensitive as human vision. The segmentation of skin lesions on RGB-colored images is difficult because of the influence of the pixel intensity [10]. Specifically, a skin lesion is likely to show different visual colors due to various conditions, such as illumination variations and low contrast between the skin lesions and a surrounding skin region. The RGB-colored images were converted to International Commission on Illumination (CIE) *L*∗*a*∗*b* color space to clearly detect the color differences between the skin lesion and the background skin. In the CIE *L*∗*a*∗*b* color space, *L* indicates the luminance (lightness) and *a* and *b* are chromaticity coordinates. The *a* axis represents a complementary color of the green-red component, and the *b* axis represents a complementary color of the blue-yellow component [22]. After color space transforming, only both of the two channels (*a* and *b*) were extracted and the lightness channel was excluded. The histogram equalization was applied to only two channels. Finally, we created a new 3-channel fusion image that reduces the illumination variations and skin color difference.

After the first step, maximum filters with a 5 × 5 kernel were also applied before the border detection to remove noise, such as hair and air bubbles. Vignetting around the image was removed by extracting the largest blobs in the binary image.

### 2.3. A Hybrid Two-Stage Segmentation Model

After preprocessing, a hybrid two-stage model was constructed for the segmentation of a melanocytic skin lesion. To obtain an initial contour mask of a melanocytic skin lesion area, the hybrid HK clustering was implemented first. Secondly, the Distance Regularized Level Set Evolution (DRLSE) was used to segment the fine border of the lesion. The detailed lesion segmentation step is described below.

#### 2.3.1. Hybrid Hierarchical *K*-Means Clustering (HK Clustering)

**Figure 4:**
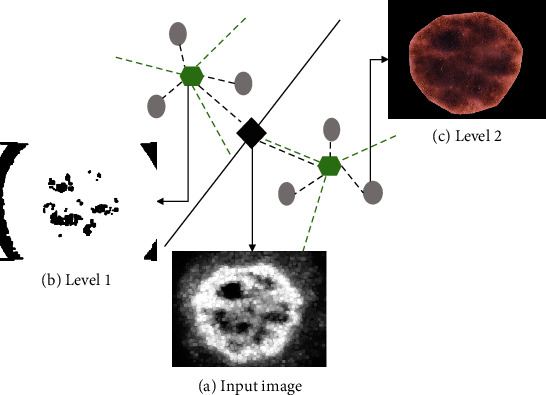
Visualization of the hybrid hierarchical *K*-means (HK) clustering method with *K*_1_ = 2 and *K*_2_ = 3 at level 1 and level 2, respectively. (a) Input image which was obtained after the preprocessing step. (b) Initial contour mask with *K*_1_ = 2 at level 1. (c) Final initial contour mask with *K*_2_ = 3 at level 2. *K* is the number of clusters at each hierarchical level.

The basic concept of HK is to recursively split the dataset into a tree of clusters with predefined branches at each node. There are two approaches to hierarchical clustering. One is the top-down technique, and the other one is the bottom-up technique [23–25]. The top-down is more efficient than bottom-up because of the fast task and greedy attributes, meaning that it cannot cross the boundaries imposed by the top level [26, 27]. In other words, nearby points may end up in different clusters. The proposed method was a modified version of the top-down approach by Chen et al. [24]. At first, the data starts as one combined cluster. Next, the cluster splits into distinct parts of *K*_1_ according to some degree of similarity (level 1). Finally, the clusters separate into distinct parts of *K*_2_ again and again until the clusters only contain some small fixed number of points (level 2).  Figure 4 shows a visualization of the hybrid HK clustering used in this study. *K*_*n*_ represents the number of clusters at the hierarchical level of *n*. The optimal number of clusters were set to *K*_1_ of 2 at level 1 and *K*_2_ of 3 at level 2 as shown in Figures 4(b) and 4(c). The number of iterations for each level of *K*-means was set to 20. The squared Euclidean distance measure was adopted for a similarity function.

#### 2.3.2. A Fine Border Segmentation Based on DRLSE Model

To segment the fine border of the melanocytic skin lesion, the DRLSE, which is one of the level set evolution approaches, was employed. The traditional level set methods consider the front as the zero-level set of an embedded function on a track moving front, called the level set function (LSF) [28–31]. The objects were detected in a given image by curve evolution [32]. To stop the curve evolution, the traditional level set method is influenced by the gradient of the given image by changing the LSF value. However, the LSF typically develops irregularities during its evolution in conventional level set formulations, which make an impact on numerical errors and eventually destroy the stability of the evolution [33]. Thus, to eliminate the need for reinitialization and avoid numerical errors, the DRLSE was employed to segment the fine border of the melanocytic skin lesions.

Each border of a skin lesion image can be regarded as the zero-level set of an LSF. Although the final segment result of the level set method is the zero-level set of the LSF, it is essential to maintain the LSF in a balanced state. This requirement can be satisfied by using signed distance functions with the unique property of |∇*ϕ*| = 1, which is referred to as the signed distance property.

Given the LSF *ϕ* : *Ω* → *ℛ* in a rectangular domain, the energy function *E*(*ϕ*) is defined by

(1) $$\begin{array}{c} E\left(f\right)=\mu R_{p}\left(f\right)+E_{\mathrm{ext}}\left(f\right), \end{array}$$

where *ϕ* is the level set function, and *R*_*p*_(*ϕ*) and *Ε*_ext_(*ϕ*) indicate the level set regularization term and external energy function, respectively. *μ* > 0 is a constant, and the level set regularization term *R*_*p*_(*ϕ*) can be defined by

(2) $$\begin{array}{c} R_{p}\left(\phi \right)≜\int_{\Omega }p\left(\left|\nabla \phi \right|\right)dx, \end{array}$$

where *p* indicates the potential function (*p* : [0, ∞] → *ℛ*). The energy *Ε*_ext_(*ϕ*) is designed to achieve a minimum value when the zero-level set of the skin lesion is located at the desired position. Moreover, the edge indicator function *g* is stated by

(3) $$\begin{array}{c} g=\frac{1}{1+{\left|\nabla \left(G_{\sigma }*I\right)\right|}^{2}}, \end{array}$$

where *I* is the image *I*(*x*, *y*) with a smoothing Gaussian kernel *G*_*σ*_, and *σ* is the standard deviation. The edge indication function stops the level set evolution when the zero-level set of the skin lesion approaches the optimal position. The energy functional *E*(*ϕ*) is determined by

(4) $$\begin{array}{c} E\left(f\right)=\mu R_{p}\left(f\right)+\lambda A_{g}\left(f\right)+\alpha B_{g}\left(f\right), \end{array}$$

where *λ* > 0 and *α* represent the coefficients of the energy functions *Α*_*g*_(*ϕ*) and *Β*_*g*_(*ϕ*), which can be written as follows:

(5) $$\begin{array}{c} A_{g}\left(\phi \right)≜\int_{\Omega }g\delta \left(\phi \right)\left|\nabla \phi \right|dx, \end{array}$$

(6) $$\begin{array}{c} B_{g}\left(\phi \right)≜\int_{\Omega }gH\left(-\phi \right)dx, \end{array}$$

where *δ* and *H* represent the Dirac delta function and the Heaviside function, respectively. Since a signed distance function is used as the initial level set function (*ϕ*_0_) in the standard level set and initialization should be done periodically to retain a stable evolution of zero level set function, the computational cost of these methods is high [34]. The level set evolution is derived as the gradient flow that minimizes an energy functional with a distance regularization term and an external energy that drives the motion of the zero-level set toward the desired location. The distance regularization term is defined by a potential function which includes a unique forward-and-backward (FAB) diffusion effect [33]. For instance, when the initial borders were located outside of the desired borders, alfa was set to a positive value to force the zero-level set to shrink toward the region of interest. In contrast, alfa was assigned a negative value to expand the borders when the initial borders were located on the inside. The detailed equation has been described previously [33].

**Figure 5:**
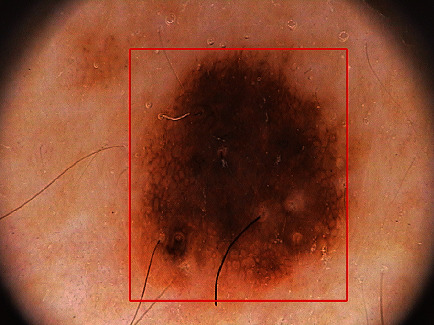
One example of the proposed method for the detection of a melanocytic skin lesion. The red rectangle indicates the boundary selection scheme, which includes the outline of a skin lesion.

**Figure 6:**
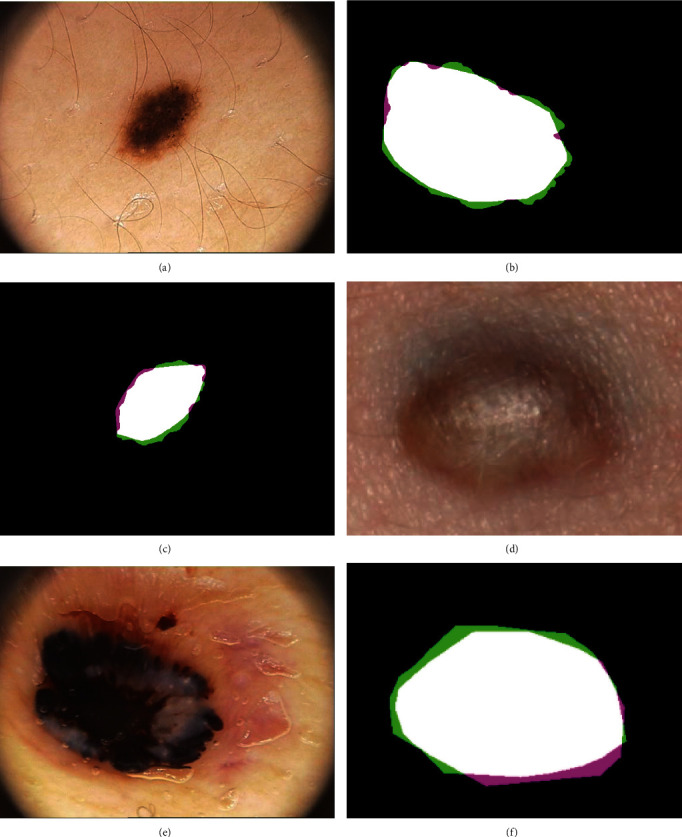
Examples of lesion segmentation results from the hierarchical *K*-means level set scheme for the PH^2^ and Dermofit atlases. (a) IMD144 dermoscopic image from the PH^2^ dataset. (c) IMD168 dermoscopic image from the PH^2^ dataset. (e) D105 dermoscopic image from the Dermofit dataset. (b, d, and f) Error evaluations: white pixels show true positives (TP), black pixels show true negatives (TN), pink pixels show false positives (FP), and green pixels show false negatives (FN).

The DRLSE parameters were set as follows: a constant controlling the gradient strength of the initial LSF(*c*_0_) of 3, a coefficient of the weighted length term (*λ*) of 5, a width of the Dirac delta function (*δ*) of 1.5, a coefficient of the distance regularization term (*μ*) of 0.02, a time-step of 8, and a standard deviation of the Gaussian kernel (*σ*) of 1.5. The initial LSF (*R*_0_) of this study was automatically detected by using the results of the HK clustering as shown in Figure 5. A set of if-then rules were applied to optimize the parameters at different conditions of images. An *α*, the coefficient of the weighted area term, was set to 3 or 5 regarding the size of the initial LSF. Double-well potential was used for a distance regularization term, and the iteration numbers were set to 600 and 1000 for the images of malignant and melanocytic nevi, respectively. A binary image was obtained with a threshold of 80. The area inside the fine border was filled in during the postprocessing step. A morphological erosion of the mask, using a square with a width of 5 pixels, and Delaunay triangulation were also carried out in the postprocessing step. Examples of the border segmentation results for the dermoscopic image (PH^2^ dataset) and standard image (Dermofit dataset) are presented in Figure 6.

### 2.4. Performance Evaluation

The output of the proposed method was binarized with a lesion mask. The performance of the proposed method was evaluated on two different datasets of melanocytic skin lesion images from the PH^2^ database [19] and the Dermofit database [20], which are publicly available on the ground truth data. To evaluate the proposed method, the well-known segmentation measures were calculated, including accuracy, specificity, sensitivity, Jaccard index (JI), Dice coefficient (DC), *F*-measure, and Hausdorff distance (HD). Specifically, these measures were calculated from the following four error factors: true positive (TP), true negative (TN), false positive (FP), and false negative (FN)

(7) $$\begin{array}{c} \text{Accuracy}=\frac{\left(\text{TP}+\text{TN}\right)}{\left(\text{TP}+\text{TN}+\text{FP}+\text{FN}\right)}, \end{array}$$

(8) $$\begin{array}{c} \text{Sensitivity}=\frac{\left(\text{TP}\right)}{\left(\text{TP}+\text{FN}\right)}, \end{array}$$

(9) $$\begin{array}{c} \text{Specificity}=\frac{\left(\text{TN}\right)}{\left(\text{TN}+\text{FP}\right)}, \end{array}$$

where TP represents the pixel numbers of a skin lesion correctly segmented as a skin lesion, TN represents the pixel numbers of background skin correctly characterized as background, FP denotes the pixel numbers of background skin incorrectly characterized as a skin lesion, FN denotes the pixel numbers of a skin lesion incorrectly characterized as background skin. Accuracy was defined as the ability to segment all areas correctly. Sensitivity was the ability to segment skin lesions. Specificity was the ability to segment the background of the skin. *F*-measure is a statistical measure of a method's accuracy that considers both the recall and the precision of the method [35]. An *F*-measure value close to 1.0 indicated that the accuracy of the proposed approach was very high. HD was calculated to measure the resemblance of two sets of points [36]. It measures how far two subsets are from each other. The smaller the HD, the greater is their degree of similarity. Additionally, the Bland-Altman plots, known as the scatter plots of the difference against the mean between the area inside the automatic border and the area inside the manual border, were also used to visualize errors and potential bias in the border detection. Furthermore, linear regression was utilized to quantitatively compare the area inside the border drawn by the two measurements. These analyses were carried out using SPSS version 23 software (SPSS Inc., Chicago, IL, USA). A *p* value < 0.05 was considered to indicate statistical significance.

The algorithm was implemented on an Intel® Core™ i5-7500 CPU at 3.40 GHz with 16.00 GB RAM. All procedures were implemented with the MATLAB software package (R2018b, MathWorks Inc., Natick, MA, USA).

## 3. Results

### 3.1. Comparison Results of Accuracy and Run-Time for Different Numbers of Clusters at Each Level (*K*_1_ and *K*_2_)

**Figure 7:**
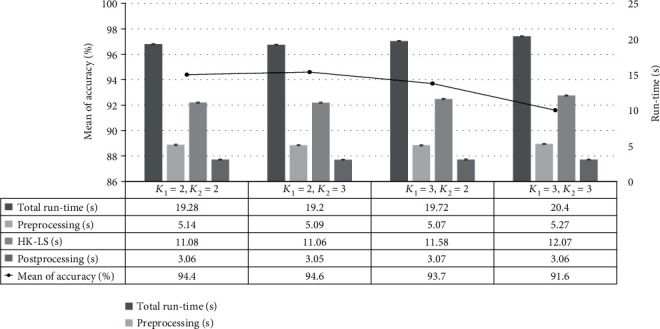
Comparison of the mean accuracy and computation time (s) for different conditions of *K*_1_ and *K*_2_ at each level (*p* value < 0.05). *K* indicates the number of clusters at each hierarchical level.

To obtain good segmentation results, the number of clusters for each level of HK clustering was experimentally determined.  Figure 7 shows the mean accuracy and speed of the proposed method in different conditions of the number of clusters from set 1 to set 4 at each hierarchical level. The run-time performance was calculated by the total time taken from the preprocessing phase to the postprocessing phase. The run-time performance for each different condition had the following relationship: set 2 (19.2 seconds) < set 1 (19.28 seconds) < set 3 (19.72 seconds) < set 4 (20.4 seconds). This suggests that set 2 outperforms other conditions in terms of run-time performance. The experimental results showed that the optimal numbers of clusters were 2 and 3 at level 1 and level 2, respectively, which achieved an accuracy of 94.6% and a speed of 19.2 seconds.

### 3.2. Quantitative Evaluation of the Proposed Two-Stage Segmentation Approach in Dermoscopic and Standard Images

The performance of the segmentation-based level set scheme depends on an initial contour mask [15, 23]. Thus, the initial segmentation is a key step to increasing sensitivity. Our method was evaluated for two different datasets as shown in Table 2. The mean accuracy for each of the two atlases was greater than 90%. The *F*-measure for each of the two datasets was high (>0.91), and a very small difference of 0.02 was found between the two atlases. Small average HDs of 0.07 ± 0.02 and 0.09 ± 0.03 were obtained for each dataset. Our method achieved higher performance in the PH^2^ database for all evaluation parameters, including sensitivity, specificity, and accuracy, than in the Dermofit database. All evaluation parameters showed promising results of over 90%, except for the Jaccard index which was 0.826 and 0.833 for the Dermofit and PH^2^ data, respectively.

### 3.3. Comparison of Results of Segmentation between Different Disease Classes (Melanocytic Nevus and Malignant Melanoma)

The proposed segmentation model was compared in two different disease classes, melanocytic nevus (common nevi, atypical nevi, and melanocytic nevi) and malignant melanoma.  Table 3 shows the segmentation results that were obtained by processing the melanocytic nevus images and melanoma images. Our method obtained good accuracy for 607 skin lesion images, including an accuracy of 93.4% for 331 images of melanocytic nevus images and 95.6% for the 76 melanoma images in the Dermofit dataset. In the PH^2^ dataset, the proposed method achieved an accuracy of 95.6% for the 160 melanocytic nevus images and 90.8% for the 40 melanoma images. Of note, the proposed method obtained a higher sensitivity of 92.6% for the melanocytic nevus images compared to a sensitivity of 86.4% for the melanoma images in the Dermofit dataset. Moreover, the *F*-measures showed 0.921 and 0.887 for the melanocytic nevus and melanoma images, respectively. In contrast, our method achieved a higher sensitivity of 92.5 for melanoma images compared to 91.7% for the melanocytic nevus images in the PH^2^ dataset. The *F*-measures became 0.920 and 0.907 for the melanoma and melanocytic nevus images, respectively.

### 3.4. The Bland-Altman Plots and Linear Regression Analysis for the Area inside Each Border Detected Manually and by the Proposed Method

**Figure 8:**
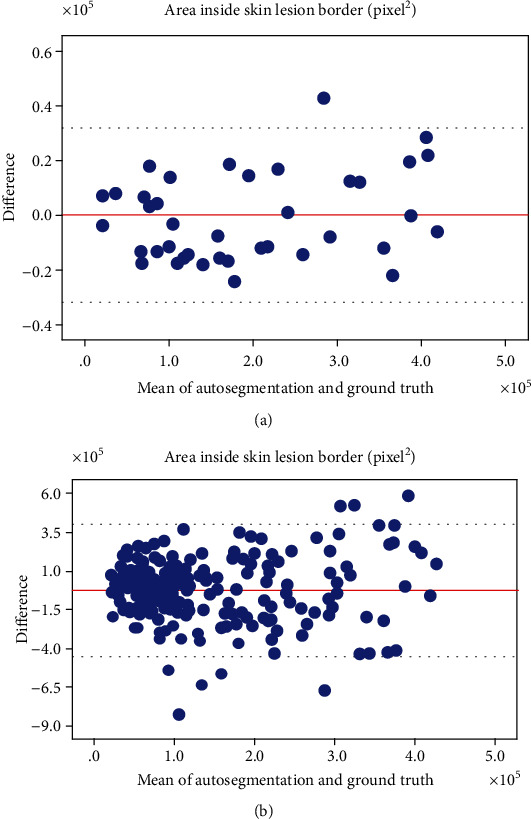
The Bland-Altman plots between the ground truth and automated segmentation of skin images obtained from the PH^2^ database. (a) Area inside the skin lesion border of the melanoma image and (b) the melanocytic nevus image (unit: pixels^2^).

**Figure 9:**
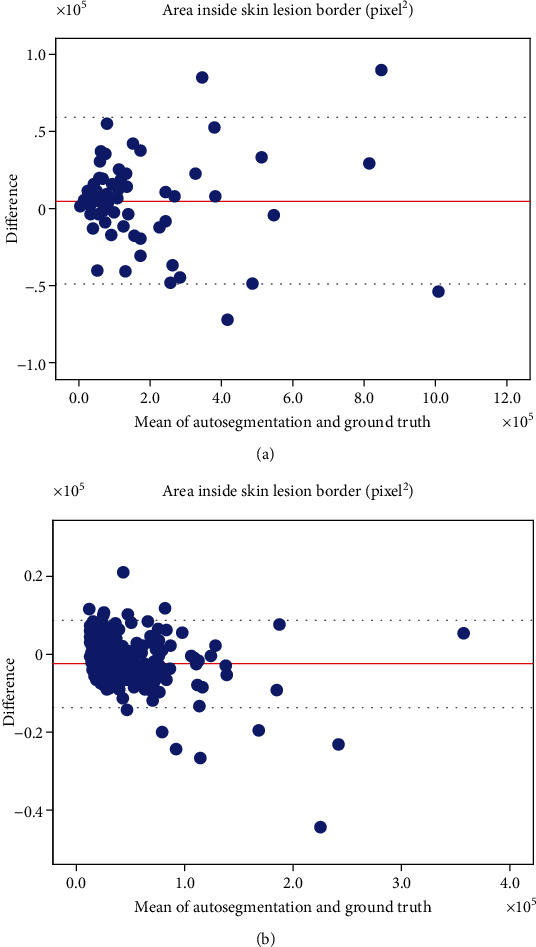
The Bland-Altman plots between the ground truth and automated segmentation of skin images obtained from the Dermofit database. (a) Area inside the skin lesion border of the melanoma image and (b) the melanocytic nevus image (unit: pixels^2^).

The mean values of the differences in the Bland-Altman plots detected by the ground truth and the proposed approach are illustrated in Figures 8 and 9. In the PH^2^ database, the average differences between the areas inside the borders detected by the ground truth and our method were 82.077 ± 15951.228 and −2702.371 ± 1498.615 for melanoma and melanocytic nevus images, respectively. In the Dermofit database, the average differences were 5025.59 ± 27,250.079 and −2279.233 ± 5734.517 for melanoma and melanocytic nevus images, respectively. All results showed differences close to 0, which were generally included within the limits of the agreement range.

**Figure 10:**
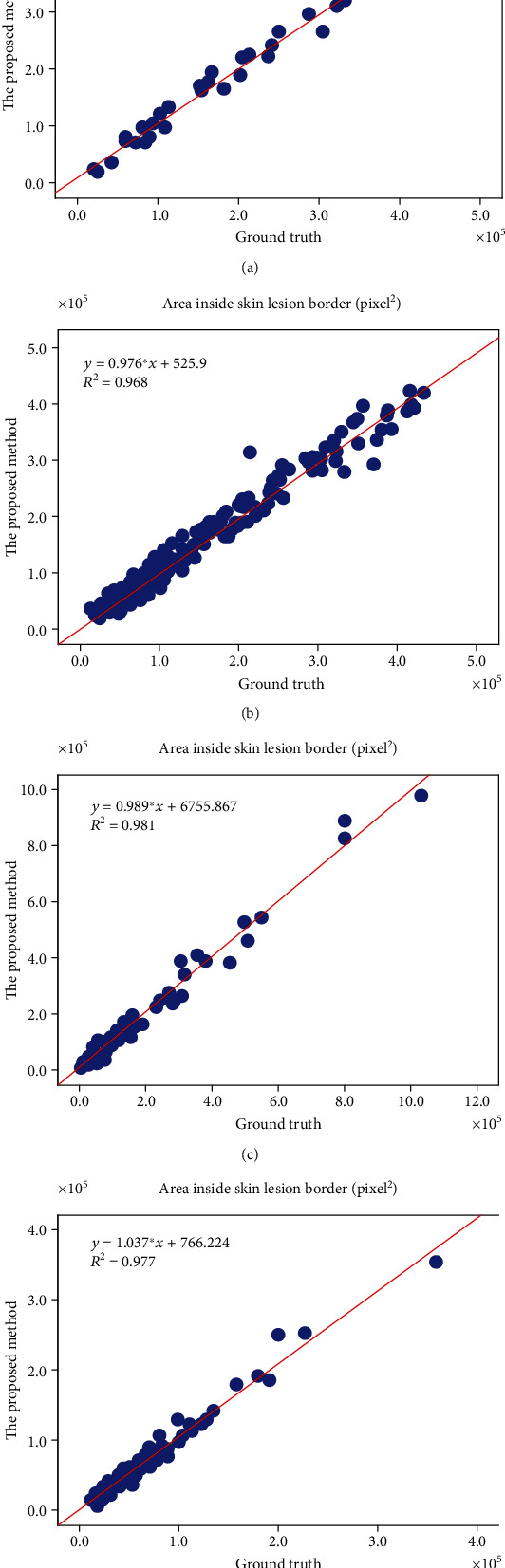
Linear regression analysis between the manual and automated segmentations of skin images obtained from the PH^2^ and Dermofit databases. (a) Area inside the skin lesion border of melanoma images from the PH^2^ database, (b) melanocytic nevus images from the PH^2^ database, (c) melanoma images from the Dermofit database, and (d) melanocytic nevi images from the Dermofit database (unit: pixels^2^).

The linear regression analysis shown in Figure 10 reports a high correlation (>0.97 and >0.96 for the Dermofit database and the PH^2^ database, respectively) between the areas inside the automated extracted borders and contours of the ground truth. These results showed that the proposed segmentation method strongly correlated with the segmentation ground truth datasets.

### 3.5. Comparison of Segmentation Performance with Other Automated Segmentation Methods

The proposed method was compared with traditional segmentation methods in the same dataset. Results of the comparison between traditional classifiers and the proposed method are summarized in Table 4. Traditional classifiers showed relatively poorer results for melanocytic lesion segmentation compared to the proposed method. Specifically, the Otsu thresholding method showed the lowest segmentation accuracies in the Dermofit and PH^2^ data (68.3% and 65.2%, respectively). The proposed method achieved a higher specificity of 94.4% than that of *K*-means clustering implemented on the same color space (CIE *L*∗*a*∗*b*). In addition, Pennisi et al. [37] segmented melanoma lesion images in the PH^2^ database using ASLM with Delaunay triangulation. They showed the accuracy of 89.7% in the PH^2^ data. In contrast, the overall accuracy of the proposed method was also better than that of the other techniques when the same dataset was used. These results demonstrated the feasibility of the proposed method for skin image segmentation.

In the comparative results between U-net [40] and our method for the PH^2^ dataset, although U-net performed better according to the Jaccard index of (0.87 ± 0.19) and Dice coefficient (0.93 ± 0.13) compared to our method, U-net produced a much larger standard deviation than that of the proposed method (Table 5). Moreover, our method had better segmentation results for the Dermofit dataset compared to that of U-net [41]. These results confirm its effectiveness for melanocytic skin lesion segmentation in standard images compared to U-net.

## 4. Discussion

The segmentation of skin lesions in dermoscopic and standard images is crucial for quantifying the clinical diagnostic factors of melanoma lesions. The segmentation accuracy can greatly affect the next diagnostic procedure [45]. One issue with the level set model is its sensitivity to the initial contours. Recently, machine learning algorithms, such as U-net, have emerged as reliable segmentation methods for skin lesion images. However, the limited training dataset is a challenging task for skin lesion segmentation. The important challenge in machine learning algorithms is that these models require a large training set to reduce overfitting. Some cases still show a low performance due to low contrast and hair artifacts. Current state-of-the-art research using machine learning algorithms is sometimes required on postprocessing techniques, such as level sets [46]. Another challenge in machine learning such as CNN is that, when a network goes deeper, it is difficult to tune the parameters of the early layers [8]. To tackle these problems, the purpose of this study was to propose a new two-stage segmentation model which integrates the Distance Regularization Level Set Evolution and the hierarchical *K*-means clustering. The proposed method that combines two different methods has the advantage of improving the final result of the image segmentation process, such as accurately defining the initial contours, and finding the approximate location of the lesion. The quantitative experimental results revealed that the proposed method yielded significantly better results compared to other traditional level set models, and has a certain advantage over the segmentation results of U-net in standard images.

The contribution of this paper can be summarized in the following aspects. Firstly, the proposed model integrates hierarchical *K*-means clustering with DRLSE. Some studies have attempted to use a mono-*K*-mean clustering-based level set evolution model with unsatisfactory results [15, 28]. However, this study showed the reliable accuracy of the segmentation of skin lesions under intrinsic noise and artifacts. To the best of our knowledge, no such studies for skin lesion segmentation have been reported previously. Secondly, the controlling parameters of level set segmentation are now derived from the results of the simple decision tree approach by using a set of if-then rules. Thirdly, the experimental results indicate that a new gray-scale image by using only the color components of *a* and *b* from CIE *L*∗*a*∗*b* color space makes it less sensitive to illumination artifacts. Finally, we also evaluated the proposed method on two different datasets including the PH^2^ database (dermoscopic image repository) and the Dermofit database (standard image repository). All skin lesions were segmented with high accuracy (>94%) and high correlation (>0.96) of the ground truth in the two databases. The segmentation results outperformed other initial estimation methods for level set models of melanoma and nonmelanoma images with various artifacts.

One of the main concerns of existing image segmentation methods resides mainly in the noise and artifacts of dermoscopic and standard images [47]. Moreover, another factor that complicates the lesion segmentation is the low contrast of the lesion boundaries [48]. Our designated model improved the segmentation performance in most cases, especially the proportion of true positive results. Experimental results show that this approach is insensitive to the low contrast between background around the lesion and skin lesion pixels. The main difference between the proposed method and other models was that only the color channels of CIE *L*∗*a*∗*b* were used to constitute a new gray-scale image for the initial contour mask. Unlike the RGB and CMYK color spaces, the CIE *L*∗*a*∗*b* is designed to approximate human vision. This color space is approximately perceptually uniform because the similarities between the perceived and the measured color are proportional [11]. Additionally, CIE *L*∗*a*∗*b* color space is known to be less sensitive to artifacts from digital cameras and scanner images [21].

When our method and other segmentation methods are compared, especially with a classifier such as U-net, our model can get better segmentation results in standard images. The latest deep learning segmentation approaches such as U-net have been applied to segment melanoma lesions because these algorithms can handle complex patterns, but the limited quality training dataset and degradation problems are often limitations [1]. In addition, data augmentation, such as flipping, rotating, shifting, scaling, and changing the contrast of the original image, is usually required when the classifier is trained on medical images [49]. However, it is easier to lose the important features of melanocytic skin lesions in data augmentation because the proportional size of the skin lesion on the images is very small [50].

Although the proposed model achieved admirable segmentation accuracy in most of the images in the two independent atlases, there were cases where the proposed model revealed the need for further improvement. The challenge in the proposed method is the increase of the run-time when the size of the image is large, compared with deep learning approaches. The proposed method can be further improved for the more effective segmentation pipeline, in terms of average run-time.

## 5. Conclusions

The segmentation of the skin lesions is regarded as very challenging because of the low contrast between the lesion and the surrounding skin, the existence of various artifacts, and different imaging acquisition conditions. The traditional model such as the region-based active contour model has often failed when applied to images containing inhomogeneities. These are very sensitive to parameter tuning. The appropriate initialization and optimal configuration of controlling parameters in the presence of various artifacts are important to obtain the accurate performance of the level set segmentation. The important challenge in machine learning algorithms is that these models require a large training set to reduce overfitting. Current state-of-the-art research using machine learning algorithms is usually required on postprocessing techniques, such as level sets. The contribution of this study is to propose a new two-stage segmentation model in dermoscopic and standard images. This method integrates a new hierarchical *K*-means and level set approach. For the initial estimation of the level set function, the hybrid hierarchical *K*-means clustering was carried out. After initial segmentation by the hybrid HK clustering, DRLSE was implemented to achieve fine border segmentation. Moreover, only the color channels of *a* and *b* from CIE *L*∗*a*∗*b* were used by this model to obtain robust image segmentation results in the presence of noise and artifacts. The generalization ability of the proposed model was validated by the independent testing of two publicly available databases. The experimental results showed the superior performance of the proposed method compared to other traditional level set models, and a certain advantage over the segmentation results of U-net in standard images. Additionally, the linear regression analysis demonstrated a good correlation of >0.98 and >0.96 with the proposed method for melanoma and melanocytic nevus images. The proposed model gives accurate segmentation results and requires a small dataset because our model is not sensitive to parameter tuning. Our experimental results revealed that integrating hierarchical *K*-means clustering and DRLSE had high clinical applicability even in the presence of various artifacts and small datasets. The proposed model may facilitate the combination of machine learning and level set models in skin lesion images.

## Figures and Tables

**Table 1:** Dataset statistics.

Atlas (the number of images)	Skin lesion	The number of images
PH^2^ data (200)	Malignant melanoma	40
	Nevus (common, melanocytic)	160
Dermofit (407)	Malignant melanoma	76
	Melanocytic nevus	331
Total (607)	Malignant melanoma	116
	Melanocytic nevus	491

**Table 2:** Quantitative evaluation of the segmentation for the Dermofit and PH^2^ atlases in terms of accuracy, sensitivity, specificity, Jaccard index, Dice coefficient, *F*-measure, and Hausdorff distance.

Group	Jaccard index	Dice coefficient	Sensitivity	Specificity	Accuracy	*F*-measure	Hausdorff distance
Dermofit	0.826 ± 0.08	0.912 ± 0.07	0.919 ± 0.08	0.944 ± 0.06	0.942 ± 0.05	0.912 ± 0.07	0.07 ± 0.02
PH^2^ data	0.833 ± 0.09	0.914 ± 0.05	0.923 ± 0.08	0.964 ± 0.05	0.946 ± 0.03	0.914 ± 0.05	0.09 ± 0.03

**Table 3:** Comparative results of segmentation between melanocytic nevus and melanoma images.

Group	Class	Jaccard index	Dice coefficient	Sensitivity	Specificity	Accuracy	*F*-measure	Hausdorff distance
Dermofit	Nevus	0.858 ± 0.08	0.921 ± 0.08	0.926 ± 0.09	0.936 ± 0.7	0.934 ± 0.04	0.921 ± 0.08	0.067 ± 0.02
	Melanoma	0.813 ± 0.08	0.887 ± 0.07	0.864 ± 0.07	0.971 ± 0.05	0.956 ± 0.06	0.887 ± 0.07	0.097 ± 0.038
PH^2^ data	Nevus	0.823 ± 0.09	0.907 ± 0.06	0.917 ± 0.09	0.978 ± 0.03	0.956 ± 0.02	0.907 ± 0.06	0.078 ± 0.03
	Melanoma	0.855 ± 0.06	0.920 ± 0.04	0.925 ± 0.05	0.845 ± 0.07	0.908 ± 0.04	0.920 ± 0.04	0.101 ± 0.05

**Table 4:** Comparative results of segmentation between the existing and proposed methods for images from the PH^2^ and Dermofit atlases.

Methods	Dermofit			PH^2^ data		
	SEN	SPE	ACC	SEN	SPE	ACC
Otsu with RGB (MATLAB 2018b)	0.611	0.723	0.683	0.522	0.706	0.652
Level set with RGB (MATLAB 2018b)	0.712	0.878	0.805	0.719	0.800	0.784
FC-LS with RGB [28]	0.873	0.926	0.918	0.891	0.914	0.904
Adaptive thresholding with YIQ [38]	0.618	0.980	0.937	0.703	0.949	0.879
*K*-means with CIELAB [21]	0.809	0.789	0.824	0.869	0.953	0.932
Local binary pattern clustering [39]	0.787	0.923	0.704	0.884	0.948	0.859
*Proposed method* (HK-LS with CIELAB)	0.919	0.944	0.942	0.923	0.964	0.946

^∗^FC-LS: fuzzy C-mean thresholding-based level set; HK-LS: hierarchical *K*-means clustering-based level set; SEN: sensitivity; SPE: specificity; ACC: accuracy.

**Table 5:** Comparative Jaccard index and Dice coefficient results for segmentation of images from the PH^2^ and Dermofit atlases by U-net and the proposed method.

Group	Dermofit		PH^2^ data	
	Jaccard index	Dice coefficient	Jaccard index	Dice coefficient
U-net [40, 41]	0.781	0.887	0.87 ± 0.19	0.93 ± 0.13
U-net with illumination-based transformation [42]	0.774 ± 0.006	0.867 ± 0.004	0.756 ± 0.009	0.853 ± 0.007
Mutual bootstrapping DCNN [43]	—	—	0.894	0.942
FCN-16s [44]			0.802	0.881
*Proposed method*	0.826 ± 0.008	0.912 ± 0.07	0.833 ± 0.09	0.914 ± 0.05

## Data Availability

The datasets that were used in this study are openly available in the PH^2^ database (https://www.fc.up.pt/addi/ph2%20database.html) [19] and the Dermofit image library (https://licensing.edinburgh-innovations.ed.ac.uk) [20].
